# Idiopathic and secondary osteonecrosis of the femoral head show different thrombophilic changes and normal or higher levels of platelet growth factors

**DOI:** 10.3109/17453674.2011.555368

**Published:** 2011-02-10

**Authors:** Elisabetta Cenni, Caterina Fotia, Enis Rustemi, Kimitachi Yuasa, Giuseppe Caltavuturo, Armando Giunti, Nicola Baldini

**Affiliations:** ^1^Laboratory for Orthopaedic Pathophysiology and Regenerative Medicine, Rizzoli Orthopaedic Institute, Bologna; ^2^Department of Human Anatomy and Musculoskeletal Pathophysiology, University of Bologna, Bologna, Italy; ^3^Department of Orthopaedic Surgery, Mie University Graduate School of Medicine, Tsu City, Mie, Japan; ^4^Immunohaematology and Transfusion Service, Maggiore Hospital, A.U.S.L. Bologna, Bologna, Italy

## Abstract

**Background and purpose:**

Thrombophilia represents a risk factor both for idiopathic and secondary osteonecrosis (ON). We evaluated whether clotting changes in idiopathic ON were different from corticosteroid-associated ON. As platelet-rich plasma has been proposed as an adjuvant in surgery, we also assessed whether platelet and serum growth factors were similar to those in healthy subjects.

**Methods:**

18 patients with idiopathic ON and 18 with corticosteroid-associated ON were compared with 44 controls for acquired and inherited thrombophilia. Platelet factor 4 (PF4), transforming growth factor-**β**1, platelet-derived growth factor-BB (PDGF-BB), and vascular endothelial growth factor were assayed in the supernatants of thrombin-activated platelets, in platelet lysates, and in serum from 14 ON patients and 10 controls.

**Results:**

Idiopathic ON patients had higher plasminogen levels (median 118%) than controls (101%) (p = 0.02). Those with corticosteroid-associated ON had significantly higher D-dimer (333 ng/mL) and lower protein C levels (129%) than controls (164 ng/mL, p = 0.004; 160%, p = 0.02). The frequency of inherited thrombophilia was not different from the controls. No statistically significant differences were found between idiopathic and corticosteroid-associated ON. 20 of the 36 ON patients were smokers. (The controls were selected from smokers because nicotine favors hypercoagulability). ON patients had significantly higher serum PF4 levels (7,383 IU/mL) and PDGF-BB levels (3.1 ng/mL) than controls (4,697 IU/mL, p = 0.005; 2.2 ng/mL, p = 0.02).

**Interpretation:**

Acquired hypercoagulability was common in both ON types, but the specific changes varied. The release of GF from platelets was not affected, providing a biological basis for platelet-rich plasma being used as an adjuvant in surgical treatment.

Osteonecrosis (ON) of the femoral head may lead to joint collapse and arthritis. In the late stages of the disease, when pain, stiffness, and disability cannot be controlled by non-surgical means, the treatment is total hip replacement. At the early stages, when less invasive treatments to preserve the femoral head such as core decompression, osteotomy, or vascularized bone grafting—or even non-invasive therapy, such as pharmacological measures, electrical stimulation, shock waves, and electromagnetic fields (Mont et al. 2007)—can prevent the affected bone from collapsing, the disease can be diagnosed only with magnetic resonance imaging. A history of one or more risk factors strengthen the suspicion of ON.

It is commonly postulated that ON is caused by reduced blood flow to the bone (Assouline-Dayan et al. 2002). Vascular occlusion and ischemia may depend on different underlying conditions such as trauma, radiation, chemotherapy, caisson disease, alcoholism, or high-dose corticosteroid therapy. However, predisposing conditions are not always evident; about 20% of cases appear to be idiopathic in origin without any associated cause (Min et al. 2008). It has been hypothesized that inherited or acquired thrombophilia and hypofibrinolysis may be risk factors for idiophatic ON (Glueck et al. 2003). However, patients with ON seldom relate having a familial or personal history of thromboembolism and the results of the specific alterations in the clotting system or of fibrinolysis are contrasting (Mehsen et al. 2009). Moreover, research has been focused on idiopathic ON without considering that thrombophilia also represents an additional risk factor for secondary ON.

On the basis of the high concentration of growth factors (GFs) released by α-granules, platelet-rich plasma (PRP) has been proposed as an adjuvant to improve angiogenesis and osteogenesis in the surgical treatment of ON (Yokota et al. 2008, Marx 2009). However, nothing is known about the levels of platelet GFs in ON.

We evaluated whether patients affected by idiopathic ON had different plasmatic and genetic abnormalities of thrombophilia and fibrinolysis from patients with corticosteroid-associated ON. We also assessed whether the platelet levels of osteogenic and angiogenic GFs were similar to those of healthy subjects.

## Patients and methods

The study protocol was approved by the institutional ethical committee on human research and was performed according to the Helsinki Declaration of 1975, as revised in 2000. The study included patients with idiopathic and corticosteroid-associated ON of the femoral head who had been diagnosed by clinical and radiological criteria and were hospitalized for hip surgery. Before surgery, an informed consent document was signed, the familial and clinical history was registered, and a sample of blood was taken. The patients had not taken heparin, oral anticoagulants, aspirin, or other platelet antiaggregants in the 2 weeks preceding blood collection. Patients with platelet disorders or platelet number less than 1 × 10^5^/μL were excluded.

18 patients had idiopathic ON and in 18 individuals the disease developed after corticosteroid treatment. Contrary to other studies, we did not find any case of alcohol-related ON. The consumption of alcohol was occasional or limited to half or one glass of wine per meal. There were no statistically significant differences in age and sex between idiopathic and corticosteroid-associated ON. The patients were compared with 44 healthy individuals who were similar to the patients regarding age and sex, and who had no clinical disorder or history of thrombosis (Table 1). A familial history of thrombosis was seldom present: there were 3 such cases with idiopathic ON and 1 such case with corticosteroid-associated ON. None of the individuals had any history of deep venous thrombosis, embolism, or myocardial infarction. Risk factors for thrombosis were seldom present: arterial hypertension (3 patients with idiopathic ON and 3 patients with corticosteroid-associated ON) and obesity (Lijnen 2009) (1 case with idiopathic ON and 2 patients with corticosteroid-associated ON). As smoking was common in patients with either idiopathic or corticosteroid-associated ON, the healthy controls were selected from a population of smokers to avoid bias based on hypercoagulability (that is favored by nicotine (Benowitz et al. 1993)) and to make a better comparison with ON patients. Staging was performed according to the radiographic criteria of the working group of the Japanese Specific Disease Investigation Committee under the auspices of the Japanese Ministry of Health, Labour and Welfare, to establish criteria for diagnosis and management of idiopathic ON of the femoral head (Sugano et al. 2002) (Table 2). There were no statistically significant differences in stage between the ON types, as analyzed with the Chi-square test.

**Table 1.** Profiles of 18 patients with idiopathic ON, 18 patients with corticosteroid-associated ON, and 44 healthy controls. For qualitative variables, the number of patients is reported

Variable	Idiopathic ON	Corticosteroid-associated ON	Healthy controls
Sex			
male	15	12	33
female	3	6	11
Age (years)			
mean (SD)	45 (12)	41 (10)	45 (10)
median (range)	41 (27–74)	42 (25–64)	44 (22–64)
Bilaterality	9	9	–
Smoking habit			
no. of smokers	13	7	42
no. of non-smokers	3	6	1
no. of unknown	2	5	1

**Table 2.** Stages of osteonecrosis (ON) in 18 patients with idiopathic ON and 18 patients with corticosteroid-associated ON, according to the radiographic criteria of the Japanese Specific Disease Investigation Committee under the auspices of the Japanese Ministry of Health, Labour and Welfare (Sugano et al. 2002). The number of patients for each stage is reported. In 1 patient with idiopathic ON, the radiographs did not show any lesions and the clinical diagnosis was confirmed by magnetic resonance imaging

	Stage				
	1	2	3A	3B	4
Idiopathic ON	0	2	7	5	3
Corticosteroid-associated ON	0	2	6	5	5

In a group of 14 ON patients (mean age 35 (19–64) years, 13 males) selected on the basis of surgery, platelet factor 4 (PF4) and GF levels in platelets and serum were compared with those in 10 healthy controls. 12 patients were affected by idiopathic ON and 2 patients by secondary ON (1 by posttraumatic ON and 1 by corticosteroid-associated ON). Age and sex were similar in the controls and patients.

### Blood collection and handling

For the clotting tests and for PF4 and GF assays, the blood was collected in 0.129 M sodium citrate. Within 2 h of collection, the samples for the clotting tests were centrifuged at 600 *g* for 10 min. The plasma was frozen in aliquots at –70°C. The samples for the assays of PF4 and GF were centrifuged at 180 *g* for 5 min in order to separate PRP from erythrocytes and leukocytes. Then the PRP was transferred to a clean tube and centrifuged at 600 *g* for 15 min. The platelets were pelleted at the bottom of the tube; platelet-poor plasma (PPP) in the upper phase was carefully transferred to a clean tube. Platelet number was adjusted to 1 × 10^6^/μL with PPP. Then the PRP was divided into 2 aliquots. In the first aliquot, platelets were lysed by 2 cycles of freeze-thawing at –80°C. In the second aliquot, the platelet release reaction was induced by adding 5 U/mL bovine thrombin (Sigma-Aldrich Corp., St. Louis, MI) for 30 min at room temperature, the samples were centrifuged, and the supernatants (from platelet release) were stored at –80°C prior to assay. PF4 and GF were also determined in serum, which was stored at –80°C after centrifugation at 600 *g* for 45 min. Blood for genetic analysis was drawn in tubes containing ethylenediaminetetraacetic acid and stored at –20°C.

### Antithrombin III

Antithrombin III was determined with a chromogenic assay (HemosIL Antithrombin, Instrumentation Laboratory, Lexington, MA). The percentage of antithrombin III activity in the samples was extrapolated from a standard curve of scalar dilutions of Calibration Plasma (Instrumentation Laboratory) according to the linear regression function.

### Protein C

Protein C activity was determined with a clotting test (HemosIL ProClot APTT-SP, Instrumentation Laboratory) based on the prolongation of APTT in the presence of activated protein C, generated by an activator derived from snake venom. The percentage of protein C activity was extrapolated by a standard curve of scalar dilutions of Calibration Plasma according to the linear regression function.

### D-dimer

D-dimer was measured by enzyme immunoassay (Asserachrom D-DI; Diagnostica Stago, Asnieres, France). The standard curve was plotted using the point-to-point method.

### Plasminogen

Plasminogen was determined with a chromogenic assay (HemosIL Plasminogen, Instrumentation Laboratory). The percentage of activity of plasminogen in the samples was extrapolated from a standard curve of scalar dilutions of Calibration Plasma according to the linear regression function.

### Plasminogen activator inhibitor-1

PAI-1 was detected in citrated plasma by enzyme immunoassay (AssayMax Human Plasminogen Activator Inhibitor-1 (PAI-1) ELISA Kit; Assaypro, St. Charles, MO). The standard curve was plotted by regression analysis using log-log curve fit.

### Platelet factor 4 and growth factor assays

PF4, transforming growth factor-β1 (TGF-β1), platelet-derived growth factor-BB (PDGF-BB), and vascular endothelial growth factor-A (VEGF-A) were determined in platelet releasates, lysates, and in serum with enzyme immunoassays according to the directions of the manufacturers (Asserachrom PF4: Stago, Asnieres, France; TGF-β1 E_max_ ImmunoAssay System: Promega Corp., Madison, WI; Quantikine Human PDGF-BB: R&D Systems Inc., Minneapolis, MN; DuoSet Human VEGF: R&D Systems). Before assay of TGF-β1, the samples were activated with 1N HCl to pH 3.0 or lower for 15 min and then neutralized with 1N NaOH to pH 7.6.

### DNA purification

Total DNA was purified from whole blood (QIAamp DNA Mini Kit; Qiagen GmbH, Hilden, Germany) and stored at –20°C.

### Factor V Leiden mutation

The single point mutation (G to A at position 1691) of the factor V gene (factor V Leiden) was detected using real-time PCR (Factor V Leiden Kit; Roche Applied Science, Monza, Italy) on the LightCycler 1.2 instrument (Roche Applied Science). The amplification program consisted of a first denaturation at 95°C for 30 seconds and 45 cycles of amplification (annealing at 55°C for 10 seconds and extension at 72°C for 5 seconds).

### Prothrombin gene mutation

The prothrombin gene mutation 20210A was detected using real-time PCR (Factor II (Prothrombin) G20210A Kit; Roche Applied Science) on the LightCycler 1.2 instrument. The amplification program consisted of a first denaturation at 95°C for 30 seconds and 45 cycles of amplification (annealing at 55°C for 10 seconds and extension at 72°C for 5 seconds).

### PAI-1 gene 4G/5G polymorphism

The PAI-1 gene 4G/5G polymorphism was detected using real-time PCR (huPlasminogen activator inhibitor 4/5G: TibMolbiol, Genoa, Italy; LC DNA Master Hybridization Probes: Roche Applied Science) on the LightCycler 1.2 instrument. Five μL of the DNA template was added to a master mix composed of 2.2 μL 4 mM MgCl2, 2 μL LC DNA Master Hybridization Probes and 1 μL of each of the primers huPAI 4/5G, in a total volume of 20 μL. The amplification program consisted of a denaturation step at 95°C for 60 seconds followed by 37 cycles of amplification (denaturation at 95°C for 1 seconds, annealing at 64°C for 10 seconds, and extension at 72°C for 13 seconds).

### Statistics

Statistical evaluations were performed using StatView for Windows version 5.0.1 (SAS Institute, Cary, NC). The statistical power was calculated with Simple Interactive Statistical Analysis (SISA) (http://www.quantitativeskills.com/sisa/). Quantitative results are reported as arithmetic mean, standard deviation (SD), median, and minimum-maximum range. Because normal distribution and homogeneity of variance were not verified by Levene's test, the differences among the groups were evaluated with Kruskal-Wallis test, and the differences between ON groups, or each ON group and the negative control, were evaluated with the Mann-Whitney U-test. The differences between the concentrations of GFs in serum and in platelet releasates or lysates were evaluated with the Wilcoxon signed rank test. The differences were considered significant at p-values < 0.05.

## Results

### Coagulation inhibitors

In corticosteroid-associated ON, protein C activity was statistically significantly lower; however, in the idiopathic type the results were similar to the healthy controls. There were no statistically significant differences between idiopathic and corticosteroid-associated ON. Only non-relevant differences were demonstrated for antithrombin III levels, either between the 2 groups of patients or between each group and the negative control (Table 3).

**Table 3.** Protein C and antithrombin III activity in 18 patients with idiopathic ON, 18 patients with corticosteroid-associated ON, and 44 healthy controls

	Protein C	Antithrombin III
	(%)	(%)
Idiopathic ON		
mean (SD)	174 (50)	110 (22)
median	163	115
minimum–maximum range	95–273	72–145
Corticosteroid-associated ON		
mean (SD)	138 (42) **^a^**	103 (17)
median	129	107
minimum–maximum range	85–243	68–125
Healthy controls		
mean (SD)	167 (35)	102 (18)
median	160	98
minimum-–maximum range	99–237	80–146

**^a^** p = 0.02 when compared with healthy controls.

### Hypercoagulability

The patients with corticosteroid-associated ON showed statistically significantly higher D-dimer concentrations than the healthy controls. In idiopathic ON, a trend towards high levels of D-dimer was observed but this was not statistically significant. No statistically significant differences were found between idiopathic and corticosteroid-associated ON (Table 4).

**Table 4.** Plasma levels of D-dimer, plasminogen, and PAI-1 in 18 patients with idiopathic ON, 18 patients with corticosteroid-associated ON, and 44 healthy controls

	D-dimer	Plasminogen	PAI-1
	(ng/mL)	(%)	(ng/mL)
Idiopathic ON			
mean (SD)	241 (132)	115 (18) **^b^**	4.6 (2.6)
median	173	118	4.7
range	86–553	72–149	1.2–10.5
Corticosteroid-associated ON			
mean (SD)	384 (274) **^a^**	104 (15)	3.6 (1.9)
median	333	101	2.9
range	85–1000	84–129	1.5–7.1
Healthy controls			
mean (SD)	173 (72)	104 (16)	3.4 (1.8)
median	164	101	3.2
range	65–338	65–129	0.6–8

**^a^** p = 0.004 vs. healthy controls

**^b^** p = 0.02 vs. healthy controls

### Fibrinolysis

The patients with idiopathic ON had statistically significantly higher plasminogen activity than the controls. The patients with corticosteroid-associated ON had plasminogen concentrations similar to the healthy controls. No statistically significant differences were found between the 2 types of ON. No significant differences in plasma concentration of PAI-1 were seen, either between the patients and the controls or when comparing the different types of ON (Table 4).

### Hypercoagulability and fibrinolysis in smokers

In smokers, D-dimer levels were significantly different in idiopathic ON, in corticosteroid-associated ON, and in healthy controls (Kruskall-Wallis test: p = 0.02), and they were increased in ON (Mann-Whitney U test: p = 0.006). In particular, smokers with idiopathic ON showed statistically significantly higher D-dimer levels than controls. In these patients, a trend was also evident regarding higher plasma PAI-1 levels than controls (Table 5). No statistically significant differences in all parameters examined were found between smokers with corticosteroid-associated ON and those with idiopathic ON or controls.

**Table 5.** Coagulation and fibrinolysis in smokers (13 with idiopathic ON, 7 with corticosteroid-associated ON, and 42 healthy controls)

	Idiopathic ON	Corticosteroid-associated ON	Healthy controls
Protein C (%)			
mean (SD)	178 (57)	152 (52)	165 (39)
median	163	148	158
range	95–273	97–243	99–237
Antithrombin III (%)			
mean (SD)	107 (22)	104 (19)	103 (19)
median	107	112.7	99
range	72–134	68–117	80–146
D-dimer (ng/mL)			
mean (SD)	227 (118) **^a^**	239 (131)	172 (73)
median	173	235	158
range	145–553	85–419	65–338
Plasminogen (%)			
mean (SD)	116 (20)	108 (18)	104 (16)
median	118	105	102
range	72–149	84–129	65–129
PAI-1 (ng/mL)			
mean (SD)	5.3 (2.5) **^b^**	3.8 (2.3)	3.4 (1.8)
median	4.8	2.6	3.2
range	1.8–10.5	2–7	0.6–8

**^a^** p = 0.01 when compared with healthy controls;

**^b^** p = 0.05 when compared with healthy controls.

### Platelet factor 4 and growth factor assays

A high degree of variability was seen in platelet and serum levels of PF4 and GFs, both in patients and in healthy controls. The levels of GFs in releasates were higher than in serum; in particular, PDGF-BB was 2.7- (2.1-) fold higher, TGF-β1 was 1.7- (2.3-) fold higher and VEGF was 1.5- (0.3-) fold higher. The lysate:serum ratio was higher than the releasate:serum ratio: for PDGF-BB it was 3.4- (1.8-) fold higher, for TGF-β1 it was 4.8- (4.5-) fold higher and for VEGF it was 1.3- (0.5-) fold higher. Even though the GF levels in releasates and lysates from ON patients were higher than those from healthy controls, no statistically significant differences were found. Instead, the serum concentrations of PF4 and PDGF-BB in ON patients were statistically significantly higher than in the controls (Table 6).

**Table 6.** Platelet factor 4 (PF4) and growth factors in releasates, platelet lysates, platelet-poor plasma (PPP), and serum in 14 patients with osteonecrosis (ON) and in 10 controls (Ctr). Releasates and lysates were obtained from platelet concentrates, after adjustment of platelet number to 1 × 10^6^ / mL

Sample	PF4 (IU/mL)		TGF-β1 (ng/mL)		PDGF-BB (ng/mL)		VEGF-A (pg/mL)	
	ON	Control	ON	Control	ON	Control	ON	Control
Releasates								
mean (SD)	7,661 (2,648)	6,856 (2,171)	55 (76)	28 (35)	8.1 (3.5)	6.4 (2.6)	165 (83)	105 (88)
median	8,265	6,939	21	16	8.1	6.5	183	105
range	4,568–10,000	3,269–10,000	2–200	2–105	2.9–13.7	2.5–11.1	9–235	9–230
Lysates								
mean (SD)	8,621 (3,231)	9,042 (1,670)	146 (85)	99 (68)	11.9 (7.4)	10.8 (6.3)	206 (180)	172 (134)
median	10,000	10,000	200	69	11.1	9.2	152	138
range	2,031–10,000	4,717–10,000	33–200	26–196	2.7–26.8	4–26	9–570	9–402
Serum								
mean (SD)	7,405 (985) ^**a**^	5,142 (1240)	38 (18)	26 (15)	4.2 (3.6) ^**b**^	2.3 (0.5)	215 (210)	182 (134)
median	7,383	4,697	50	29	3.1	2.2	155	160
range	5,777–8,553	3,576–7,165	14.6–50	2.5–50	1.1–13.5	1.3–3.1	9–628	9–493

^**a**^ p = 0.005 when compared with controls;

^**b**^ p = 0.02 when compared with controls.

### Heritable thrombophilia and hypofibrinolysis

Homozygosity or heterozygosity for factor V Leiden was present in only 2 patients affected by corticosteroid-associated ON. Factor V Leiden was not detected in any patients with idiopathic ON or in any controls. Homozygosity for the prothrombin gene mutation was not found in any patients or controls; heterozygosity was present in only 2 patients with idiopathic ON and in 1 control. The genotypes 4G/5G and 4G/4G of the PAI-1 gene were demonstrated in 7 and 6 of 18 patients with idiopathic ON, and in 9 and 4 of 18 patients with corticosteroid-associated ON. The frequency of 4G/4G homozygosity was also high in healthy controls (19/44), while 16 of the 44 control subjects were heterozygous.

## Discussion

We hypothesized that the alterations in the clotting system or in fibrinolysis in idiopathic ON may be different from those in corticosteroid-associated ON. The secondary hypothesis was that the levels of GFs in ON platelets are similar to or higher than in those of healthy subjects, thus supporting the use of PRP as an adjuvant for the treatment of ON.

Although the idiopathic and corticosteroid-associated ON groups were homogenous regarding age, sex, and stage, one limitation of the study may be the low number of cases. The 2 types of ON showed different alterations in the clotting tests. In particular, higher levels of plasminogen were detected in idiopathic ON while lower protein C activity and higher concentrations of D-dimer were found in corticosteroid-associated ON.

The high plasminogen levels in idiopathic ON may have several explanations. Some degree of synovial inflammation has been shown in ON and it was hypothesized to play a role in the disease progression (Rabquer et al. 2009). Plasminogen, which increases in phlogosis, could be a systemic marker of the inflammatory state present in ON, such as erythrosedimentation rate, which has been found to be elevated in 78% of ON patients (Séguin et al. 2008). Also, the trend toward higher levels of PAI-1—which was particularly evident in smokers with idiopathic ON—may have contributed to the increase in plasminogen. In fact, PAI-1 inhibits tissue plasminogen activator and therefore generation of plasmin from plasminogen. This trend of increased PAI-1 levels was only partially explained by homozygosity for the 4G/4G polymorphism of the PAI-1 gene, which leads to higher transcription of the gene and therefore higher production of PAI-1 protein (Asano et al. 2004). Notably, in our patients the frequency of homozygosity for the 4G/4G polymorphism was lower than in the healthy controls, and was also lower than the 41% reported by Glueck et al. (1999) for ON. Inflammation (Aso 2007, Kruithof 2008) or endothelial activation (Salgado et al. 1994) may be another possible explanation for the increase in PAI-1. Endothelial activation, which has been reported in ON (Séguin et al. 2008), could have caused the increase in PAI-1 in our patients. One possible limitation of our data was the low sample number. The statistical power analysis based on idiopathic ON-control differences in plasminogen revealed a power of 62% for α = 0.05. Thus, these results should be considered preliminary and they require confirmation.

Normal or low levels of plasminogen have been reported by other authors in both ON and Perthes' disease, which has a similar pathogenesis. Lee et al. (2003) did not show any variation between non-traumatic ON patients and controls. In Perthes' disease, reduced plasminogen was reported by Pósán et al. (2003), but normal levels were reported by Koo et al. (2002). These discordant results may depend on ethnic differences, as hypothesized by Lee et al. (2003). On the other hand, contrasting data have been reported in the literature for other clotting or fibrinolytic alterations in ON, and in Perthes' disease. Probably different thrombophilic changes, but all drivers to hypercoagulability, may be involved in ON pathogenesis among different ethnic groups or even among different individuals.

For all the parameters examined, no statistically significant differences between idiopathic and corticosteroid-associated ON were found. Corticosteroid-associated ON showed peculiar alterations, consisting of a decrease in protein C and in an increase in D-dimer relative to healthy controls. A possible limitation of the finding of a reduction in protein C in corticosteroid-associated ON was the fact that the statistical power was 72% for α = 0.05; thus, our findings require to be confirmed by further studies. Other authors have found reduced protein C activity in non-traumatic ON (Zalavras et al. 2000). In Perthes' disease, discordant results have been reported for protein C: some authors have not found any changes (Almeida Matos 2008) while others have found reduced levels (Mehta et al. 2006).

Instead, the statistical power analysis based on difference between corticosteroid-associated ON and controls regarding D-dimer levels revealed a power of 95% for α = 0.01. An increase in D-dimer was reported in 25% and 21% of patients with idiopathic or secondary ON, respectively, as a consequence of the hypercoagulability that also induced ON (Pósán et al. 2003). Corticosteroid treatment did not affect D-dimer levels in healthy volunteers (Brotman et al. 2006), but induced thrombosis and ON in rabbits, when associated with a low dose of lipopolysaccharide (Wu et al. 2008). Even though glucocorticoids may increase PAI-1 gene transcription (Halleux et al. 1999) and plasma levels (Kerachian et al. 2009) in corticosteroid-associated ON, PAI-1 concentration was similar to that in the healthy controls, probably because the patients were not taking corticosteroids when they were enrolled.

We found a low frequency of inherited thrombophilia both in idiopathic ON and corticosteroid-associated ON. The frequencies of factor V Leiden and prothrombin gene mutations were similar to those in the controls, contrary to what has been asserted by some authors who have hypothesized that idiopathic ON might be favored by factor V Leiden or prothrombin gene mutation (Björkman et al. 2004).

Interestingly, 55% of the ON patients were smokers, as compared to a rate of 23% in the general population (Activities for the prevention of smoking—2009 report, Ministry of Health, Italy, http://www.salute.gov.it/imgs/C_17_pubblicazioni_1161_ulteriorallegiati_ulterioreallegato_0_alleg.pdf). Other researchers have found a strong association between tobacco consumption and risk of ON. Mehsen et al. (2009) found a frequency of 69% smokers in ON patients and a frequency of 38% smokers in controls. Sakaguchi et al. (2010) observed that more patients who developed ON after corticosteroid therapy had a smoking habit (68%) than controls (50%). Also, in Perthes' disease an association between the femoral lesion and smoking by the mother during pregnancy or passive smoking after birth has been demonstrated (Glueck et al. 1998). Nicotine has been reported to cause vasoconstriction (Winniford et al. 1987) and to induce bovine and human endothelial cells to produce PAI-1 (Kuo et al. 1989, Zidovetzki et al. 1999). Increased plasma levels of D-dimer (Wannamethee et al. 2005) and PAI-1 (Tapson 2005) have been found in healthy smokers.

In our study, D-dimer levels were higher in smokers with ON than in smokers in the control group. This result was more evident in smokers with idiopathic ON. In these patients, a trend of higher plasma levels of PAI-1 was also found. Thus, we could hypothesize that smoking is only one of several risk factors for idiopathic ON. Other causes may induce hypercoagulability, which is important for ON pathogenesis. In smokers with corticosteroid-associated ON, D-dimer levels were not statistically significantly different from those in the controls, which is contrary to what was observed when we considered smokers and non-smokers together. In this group, other causes, independent of smoking and present also in non-smokers, could have induced the increase in D-dimer. Mehsen et al. (2009) did not consider the laboratory tests of ON patients and controls separately according to their smoking habits. Sakaguchi et al. (2010) did not perform any laboratory test because they considered only anamnestic risk factors for corticosteroid-associated ON. On the basis of the above considerations, smoking may be considered to be more of a possible risk factor when associated with other causes than an etiological factor of ON, through its proinflammatory, procoagulant, antifibrinolytic, and antiendothelial effects (Yanbaeva et al. 2007).

In order to establish a scientific basis for the use of platelet-rich plasma in ON, we determined the levels of some osteogenic and angiogenic GFs in platelet lysates and releasates. The levels of platelet GFs are known in healthy blood donors (Zimmermann et al. 2003), but the effects of bone diseases such as ON on the concentrations are unknown. As GF levels in plasma originate from different cell types, we determined also PF4, which is specific for platelet α-granules (Kaplan 1978). In releasates, PDGF-BB, TGF-β1, and VEGF originated from platelet activation induced by thrombin and their levels were lower than in lysates, which contained the total amount present in platelets. Some of the GFs were entrapped in the fibrin network and were slowly released during clot retraction. The levels of GFs in ON were similar to or higher than in healthy controls. These results support the use of PRP as an adjuvant in ON treatment. The higher concentrations of PF4 and PDGF-BB in the serum of ON patients may be due to the increased platelet activation associated with the thrombophilic state (Pósán et al. 2003).

In conclusion, we did not detect statistically significant differences in clotting tests between idiopathic and corticosteroid-associated ON. In comparison with healthy controls, idiopathic ON showed higher plasminogen activity; corticosteroid-associated ON had lower protein C levels and higher D-dimer levels. However, these results require to be confirmed by further studies on a larger number of patients, particularly the data regarding plasminogen and protein C. In both groups of patients, the frequency of heritable thrombophilia and fibrinolysis was similar to that for the controls. Thus, the results of this preliminary study support the idea that both idiopathic and corticosteroid-associated ON are favored by acquired hypercoagulability, but at a subclinical level. The high levels of PF4 in releasates showed that platelet release reaction is not affected in ON. The concentrations of GFs in releasates and lysates from ON patients were similar to those in healthy subjects. This result provides a biological basis for the use of PRP in ON treatment.
